# Antitumor effects of anlotinib in thyroid cancer

**DOI:** 10.1530/ERC-17-0558

**Published:** 2018-08-21

**Authors:** Xianhui Ruan, Xianle Shi, Qiman Dong, Yang Yu, Xiukun Hou, Xinhao Song, Xi Wei, Lingyi Chen, Ming Gao

**Affiliations:** 1 Department of Thyroid and Neck TumorTianjin Medical University Cancer Institute and Hospital, National Clinical Research Center for Cancer, Key Laboratory of Cancer Prevention and Therapy, Tianjin, China; 2 State Key Laboratory of Medicinal Chemical BiologyKey Laboratory of Bioactive Materials, Ministry of Education, Collaborative Innovation Center for Biotherapy, Tianjin Key Laboratory of Protein Sciences, 2011 Collaborative Innovation Center of Tianjin for Medical Epigenetics and College of Life Sciences, Nankai University, Tianjin, China; 3 Department of Diagnostic and Therapeutic UltrasonographyTianjin Medical University Cancer Institute and Hospital, National Clinical Research Center of Cancer, Key Laboratory of Cancer Prevention and Therapy, Tianjin, China

**Keywords:** anlotinib, multi-kinase inhibitor, thyroid cancer, apoptosis, migration

## Abstract

There is no effective treatment for patients with poorly differentiated papillary thyroid cancer or anaplastic thyroid cancer (ATC). Anlotinib, a multi-kinase inhibitor, has already shown antitumor effects in various types of carcinoma in a phase I clinical trial. In this study, we aimed to better understand the effect and efficacy of anlotinib against thyroid carcinoma cells *in vitro* and *in vivo*. We found that anlotinib inhibits the cell viability of papillary thyroid cancer and ATC cell lines, likely due to abnormal spindle assembly, G2/M arrest, and activation of TP53 upon anlotinib treatment. Moreover, anlotinib suppresses the migration of thyroid cancer cells *in vitro* and the growth of xenograft thyroid tumors in mice. Our data demonstrate that anlotinib has significant anticancer activity in thyroid cancer, and potentially offers an effective therapeutic strategy for patients of advanced thyroid cancer type.

## Introduction

Thyroid cancer is the most common cancer of endocrine system, with a rapid worldwide increase over recent decades (Enewold* et al.* 2009, Kilfoy* et al.* 2009, Chen* et al.* 2016). The disease is classified into three types based on pathological characteristics: papillary carcinoma (PTC), follicular carcinoma (FTC) and anaplastic carcinoma (ATC) (Cho* et al.* 2013). About 90% of thyroid cancer are well differentiated, while 10% or less are poorly differentiated or anaplastic subtypes (Kondo* et al.* 2006, Xing 2013). Of the differentiated carcinomas, 85–90% are PTC and 10–15% are FTC (Baudin & Schlumberger 2007). ATC is a rare, but very aggressive, human malignant tumor. The approximate incidence of ATC is one to two cases per million every year, but the median survival of ATC patients is only about five months (Nagaiah *et al*. 2011, Smallridge* et al.* 2012). Most thyroid cancer patients become disease-free after initial treatment with surgical resection, radioiodine, and thyroid hormone therapy (McFarland & Misiukiewicz 2014). However, there are few treatment options available for patients with advanced disease, including radioiodine-resistant and metastatic differentiated thyroid cancer and anaplastic thyroid cancer (ATC). Tumors initially categorized as poorly differentiated thyroid cancer (PDTC) or ATC are often highly aggressive and recurrent. In addition to their aggressive growth and metastasis, loss of the capacity to uptake iodine makes both PDTC and ATC difficult to treat, leading to poor prognosis (Smallridge* et al.* 2009, McFarland & Misiukiewicz 2014). Moreover, chemotherapeutic treatment has been proved to be largely ineffective against aggressive thyroid carcinomas. These inadequacies of current treatment protocols for PDTC and ATC strongly emphasize the urgent need for novel targeted treatment options (Sherman 2009).

Over the past few decades, significant advances have been made in the understanding of the molecular pathogenesis of thyroid cancer (Xing 2013). The pathogenesis of thyroid cancer is thought to involve a multi-step process, in which genetic alterations in oncogenes and tumor suppressor genes lead to aberrant proliferation of cells, and alterations in angiogenic genes lead to tumor invasion and spread (Fagin & Mitsiades 2008). Some important tumorigenic factors have been identified as potential therapeutic targets for novel anticancer treatments. Multi-targeted tyrosine kinase inhibitors have demonstrated significant antitumor effects in a variety of tumor types, including thyroid cancer, by inhibiting the angiogenic and proliferative signaling (Lorusso* et al.* 2016). Recently, some kinase inhibitors such as sorafenib, vandetanib and cabozantinib have been proved to be the first-line therapies of advanced thyroid malignancies. In addition, more and more multi-kinase inhibitors are included in clinical trials (Covell & Ganti 2015).

Anlotinib is a new multi-kinase inhibitor that has shown efficacy against a wide variety of tumors in preclinical models. It has been reported that anlotinib is safe and efficient to treat patients with advanced refractory solid tumors (Sun* et al.* 2016). Anlotinib suppresses tumor cell proliferation and angiogenesis, via inhibition of platelet-derived growth factor receptor, Ret, Aurora-B, epidermal growth factor receptor and fibroblast growth factor receptor (FGFR) (Wang* et al.* 2016).

The purpose of the studies reported here was to investigate the antitumor efficacy and mechanism of anlotinib in preclinical models of PTC and ATC. Three PTC cell lines and three ATC cell lines were used to elucidate the effects of anlotinib at different doses on proliferation. The IC_50_ of anlotinib on these cells range from 3.02 to 5.42 µM. We found that anlotinib inhibits the cell viability of thyroid cancer cells, and arrests cells at the G2/M phase, most likely due to abnormal spindle assembly, but not the BRAF/MEK/ERK pathway, one of the most important signaling pathways in thyroid cancer. Cell apoptosis assay revealed that anlotinib induces apoptosis of thyroid cancer cells, partly through activating the TP53 pathway. Anlotinib also inhibits the migration of thyroid cancer cells, through interfering F-actin formation. In addition, anlotinib suppresses the growth of xenograft thyroid tumors in mice. These data provided the first evidence that anlotinib may have a high therapeutic efficacy in thyroid cancer, as both antitumor and antimetastatic agents.

## Materials and methods

### Compounds

Anlotinib was kindly provided by Tai Tianqing (Nanjing, China). PD0325901 was purchased from Sigma. Compounds were dissolved in dimethyl sulfoxide (DMSO, Sigma), and diluted with culture medium to the desired concentration for *in vitro* studies.

### Cell lines and cell culture

Three PTC cell lines, BCPAP, K1, and IHH4, and three ATC cell lines, 8505C, CAL-62, and BHT-101, were used in this study. K1, IHH4 and 8505C were kindly provided by Prof. Haixia Guan (China Medical University, China) and all other cell lines were purchased from American Type Culture Collection. BCPAP, K1, IHH4, and 8505C cells were cultured in growth medium consisting of 90% RPMI-1640 (Gibco), 10% FBS, 2 mM l-glutamine, 5000 units/mL penicillin and streptomycin. CAL-62 and BHT-101 cells were cultured in growth medium consisting of 85% DMEM (high glucose, Invitrogen), 15% FBS, 2 mM l-glutamine, 5000 units/mL penicillin and streptomycin. All the cell lines were authenticated with Short Tandem Repeat DNA profiling analysis routinely, BRAF mutational status of BCPAP, K1 and 8505C were verified by sanger sequencing. The passage number of the cells used for the experiments was about 20–30.

### Cell viability assay

Cell viability was measured using MTT (Sangon, China). Cells (5 × 10^3^/well) were seeded into 96-well plate and incubated for 24 h. Then 100 µL medium containing 0, 0.01, 0.1, 0.4, 0.8, 1, 4, 8, 10, 15 and 20 µM anlotinib were added into each well. After 72 h anlotinib treatment, cells were incubated with MTT substrates (5 mg/mL) for 4 h. Culture medium was removed, and DMSO was added. Optical density was measured at 570 nm. Calculation of results and Student’s *t*-test were performed using SoftMax pro software (Molecular Devices), and IC_50_ values were calculated using Sigma Plot software (Systa, San Jose, CA, USA).

### Cell cycle analysis

Cells were treated with DMSO or 10 µM anlotonib for 24 h. Treated cells were harvested, fixed in cold 70% ethanol, and incubated at 4°C overnight. Cells were resuspended in PBS buffer supplemented with 60 µg/mL RNase A and 25 µg/mL propidium iodide (PI), and incubated 20 min in dark at 37°C. Samples were analyzed with a FACS Calibur flow cytometer (BD Biosciences).

### Cell apoptosis analysis

Cells were treated with DMSO or 10 µM anlotinib for 36 h. Cells collected by trypsinization and washed with PBS. To detect cell death, an Annexin V apoptosis detection kit (Beyotime) was used. Briefly, 1 × 10^5^ cells were mixed with Annexin V-FITC and PI, and incubated for 15 min at room temperature in the dark. Stained cells were analyzed with a FACS Calibur flow cytometer (BD Biosciences).

### Antibodies and Western blotting

Cells were lysed in lysis buffer (Beyotime), and protein concentration was measured using BCA Protein Assay kit (Beyotime) to ensure equal loading. The samples were resolved by SDS-PAGE, followed by transferring onto a PVDF membrane (Millipore).

Membranes were probed with primary antibodies. Bound primary antibodies were recognized by HRP-linked secondary antibodies (GE Healthcare). Immunoreactivity was detected by ECL Plus (Beyotime) and Kodak light film. Digital images of films were taken with BioRad Molecular Imager Gel Doc XR. Primary antibodies used for Western blot and immunohistochemistry are TP53 (Cell Signaling Technology), Cl-caspase 3 (Cell Signaling Technology), Cl-PARP (Cell Signaling Technology), TUBULIN (Huada), p-ERK (Cell Signaling Technology), ERK (Cell Signaling Technology), p-MEK (Cell Signaling Technology), MEK (Cell Signaling Technology), Ki67 (Abcam).

### RNA interference

Small interfering RNAs (siRNA) against TP53 and control RNA were purchased from Sigma. The small siRNA target TP53 (EHU123221) was purchased from Sigma. All siRNA transfections were performed using Lipofectamine3000 Transfection Reagent (Invitrogen) following the manufacturer’s recommendations. The final concentration of the siRNA molecules is 10 nM and cells were treated with anlotinib 24 h later.

### Immunoﬂuorescence assay

Cells were ﬁxed in 4% paraformaldehyde for 20 min, and then permeabilized with 0.2% Triton X-100 for 30 min. After being blocked with 5% goat serum for 2 h, cells were incubated with primary antibodies for 4–6 h at room temperature or overnight at 4°C. Then cells were washed and incubated with secondary antibodies and/or rhodamine-phalloidin (Molecular Probe). Alexa Fluor 488 anti-mouse was used as secondary antibodies (Molecular Probe), and Hoechst 33342 (Sigma) for nuclei staining. Epiﬂuorescent images were taken with Olympus IX81 microscope. Confocal images were captured using Leica TCS SP5 confocal microscope.

### Wound healing assay

Cells were seeded into 6-well plate and grown to a confluent monolayer. A line was scratched on the cell monolayer with a p200 pipette tip. The plates were washed with PBS to remove detached cells, and then incubated with complete growth media containing 0, 1, 5 or 10 µM anlotinib for 12 h. Cell migration was observed under a phase-contrast microscope at 40× magnification at 0 and 12 h post-induction of injury. Migration distance was analyzed using ImageJ software. Four pairs of images were analyzed for each sample.

### Colony formation assay

For colony forming assay, cells were seeded in a 6-well plate at 300 cells per well in duplicates. Twenty-four hours later, cells were cultured with media containing 0, 1, 5 or 10 µM anlotinib for 8 days. Cells were then stained with 0.05% crystal violet before photographing.

### *In vivo* tumorigenicity assay

All of the experimental protocols and animal care were approved by the Ethics Committee of the Tianjin Medical University Cancer Institute and Hospital, and were in compliance with the principles and procedures outlined in the NIH Guide for the Care and Use of Laboratory Animals. K1 cells were harvested and resuspended at 1 × 10^7^ cell/mL with phosphate-buffered saline. Groups of 4-week-old male BALB/c athymic nude mice (each group, *n* = 5) were subcutaneously injected at the buttock with 0.1 mL of the suspension. Tumor growth was measured on day 6 after injection, and then every 3 days. One group were intraperitoneally treated with 3 mg/kg anlotinib, once daily for two weeks. Another group were treated with equivoluminal DMSO, once daily for 2 weeks. At the indicated time points, the animals were killed and the tumors were excised and measured.

### Haematoxylin and eosin staining and immunohistochemistry (IHC)

Dissected tumor tissues preserved in 4% PFA at 4°C for 24 h, dehydrated through xylenes and alcohols, and embedded in paraffin. Sections were cut at 5 μm and stained with haematoxylin and eosin (HE) for histological examination. Immunohistochemistry was performed according to standard protocols. Signal was visualized with DAB Substrate Kit (MaiXin Bio). Images were taken with Leica DM3000 Microscope.

### Statistical analysis

All data were analyzed by Student’s *t*-test. Statistically significant *P* values were indicated in figures as follows: ***P < 0.001, **P < 0.01, *P < 0.05.

## Results

### Anlotinib inhibits the proliferation of PTC and ATC cells

**Figure 1:**
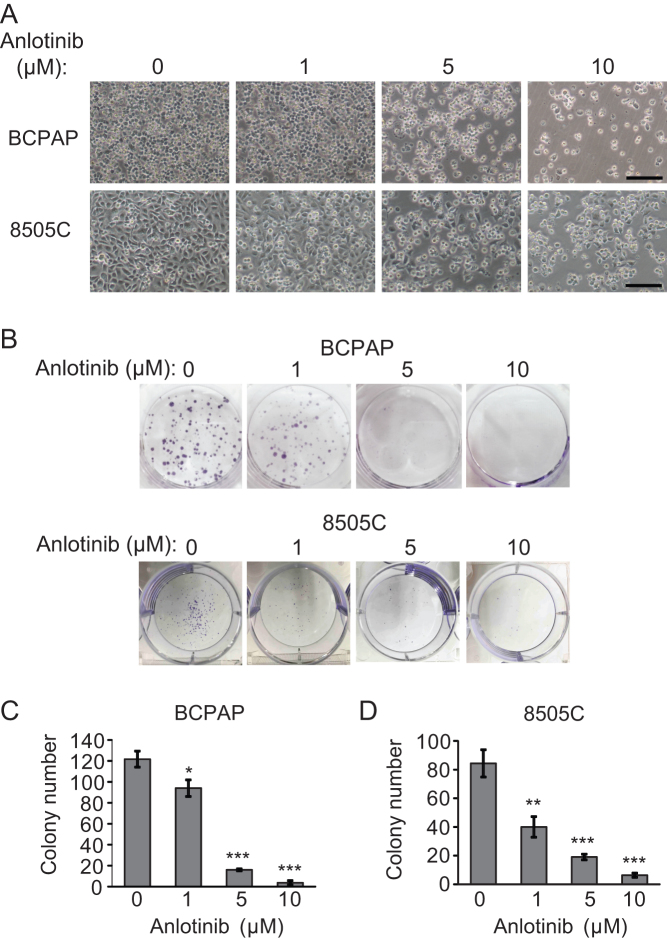
Anlotinib inhibits thyroid cancer cells proliferation *in vitro*. (A) Thyroid cancer cells BCPAP and 8505C were treated with anlotinib (0, 1, 5 and 10 µM) for 24 h, and then photographed with inverted contrast microscopy (100× magnification). Scale bars: 50 µm. (B) Representative images of colony formation assays using BCPAP and 8505C cells treated with (0, 1, 5 and 10 µM) for 8 days. (C and D) Quantification of the colony formation assays in BCPAP and 8505C cells. Data are shown as mean ± s.d. (*n* ≥ 3).

To assess whether anlotinib inhibits the proliferation of cells derived from different thyroid tumor subtypes, we treated BCPAP (a PTC cell line) and 8505C (an ATC cell line) with anlotinib at increasing concentrations (0, 1, 5 and 10 µM) for 24 h. The morphologic changes of thyroid cancer cells were examined under a phase-contrast microscope. The number of cells is dramatically reduced with the increasing concentration of anlotinib. Cells start to shrink, lose their normal shape, become round and ultimately detach from the culture dish (Fig. 1A). Next, we asked if the antitumor effect of anlotinib is ubiquitous for various thyroid cancer subtypes. Three PTC lines (BCPAP, K1 and IHH4) and three ATC lines (8505C, CAL-62 and BHT-101) were treated with anlotinib at different concentrations, and then subjected to the MTT assay. The viability of cells in all six thyroid cancer cell lines were all reduced when the concentration of anlotinib was greater than 1 µM (Supplementary Fig. 1A, see section on supplementary data given at the end of this article). IC_50_ for all six cell lines range from 3.02 µM to 5.42 µM (Supplementary Fig. 1B). These data suggest that anlotinib effectively inhibits the cell viability of both PTC and ATC cells. Overall, PTC cells are slightly more sensitive to anlotinib than ATC cells.

To further validate the anti-proliferative effects of anlotinib on thyroid cancer cells, colony formation assay was carried out with BCPAP and 8505C cells. Twenty-four hours after seeding, BCPAP and 8505C cells were treated with anlotinib at various concentrations (0, 1, 5 and 10 µM) for 8 days. The number and the size of colonies were measured on day 9. As the concentration of anlotinib increases, the number of colonies decreases, and the size of colonies becomes smaller (Fig. 1B, C and D). These data confirm that anlotinib elicits an anti-proliferative effect on BCPAP and 8505C cells in a dose-dependent manner.

### Anlotinib induces G2/M arrest and abnormal spindle assembly in thyroid carcinoma cells

**Figure 2:**
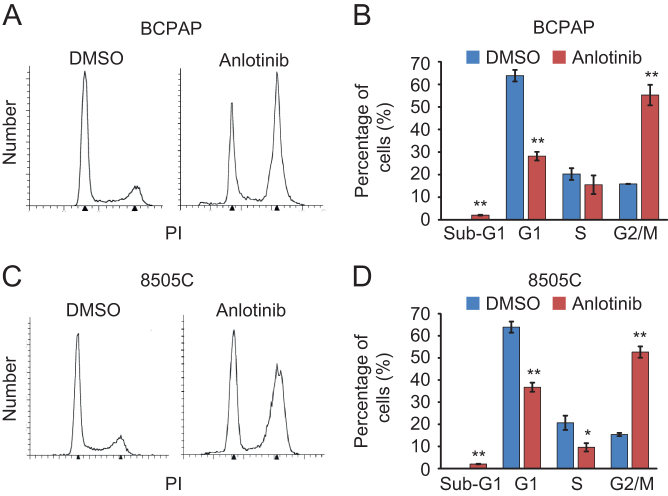
Anlotinib induces G2/M phase arrest in thyroid cancer cells. (A and C) BCPAP cells (A) and 8505C cells (C) were treated with DMSO or 10 µM anlotinib for 24 h and stained with PI. Cell cycle distribution was assessed with flow cytometry and quantified by Modifit software. (B and D) The percentage of cells in sub-G1, G1, S and G2/M phase were calculated and plotted. Data are shown as mean ± s.d. (*n* ≥ 3).

Given the anti-proliferative effect of anlotinib on thyroid cancer cells, we asked whether anlotinib inhibits cell proliferation by regulating cell cycle progression. BCPAP and 8505C cells were treated with or without 10 µM anlotinib for 24 h. The cells were then stained with PI and analyzed by flow cytometry. The fraction of G2/M cells increases markedly in anlotinib-treated BCPAP and 8505C cells. In addition, anlotinib treatment also leads to a slight enhancement of sub-G1 fraction, indicating that anlotinib induces cell apoptosis as well (Fig. 2).

The activation of the BRAF/MEK/ERK pathway plays an important role in thyroid cancer (Espinosa* et al.* 2007, Girotti & Marais 2013). The *BRAFV600E* mutation is found in approximately one-half of PTC patients and one-fourth of ATC cases and is associated poor prognosis (Li* et al.* 2012, Liu* et al.* 2016, Xing* et al.* 2013). We first tested whether anlotinib inhibits cell proliferation and induces G2/M arrest by blocking the BRAF/MEK/ERK signaling pathway. To evaluate the effect of anlotinib on the BRAF/MEK/ERK pathway in BCPAP and 8505C cells, the phosphorylation levels of MEK and ERK were determined by Western blot. Only at high concentrations (10 and 15 µM), anlotinib reduces the phosphorylation levels of MEK and ERK in BCPAP cells (Supplementary Fig. 2A). In contrast, MEK and ERK phosphorylation in 8505C cells is not affected by anlotinib, even at the highest concentration tested (Supplementary Fig. 2B), suggesting that the anti-proliferative effect of anlotinib is not mediated by the suppression of the BRAF/MEK/ERK pathway, at least in 8505C cells. Moreover, a specific MEK inhibitor PD0325901 (PD), which effectively blocks the MEK/ERK pathway, has little effect on the cell viability of BCPAP and 8505C cells (Supplementary Fig. 2). The above data showed that the anti-proliferative effect of anlotinib on thyroid cancer cells is not through inactivating the BRAF/MEK/ERK pathway.

**Figure 3:**
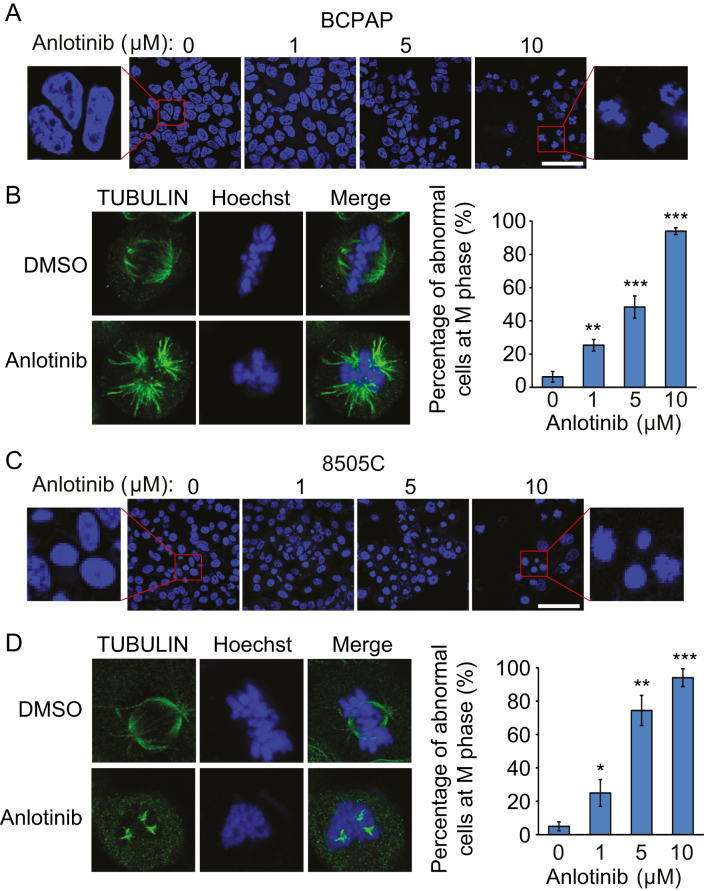
Anlotinib leads to abnormal spindle formation in thyroid cancer cells. (A and C) BCPAP cells (A) and 8505C cells (C) were treated with anlotinib (0, 1, 5 and 10 µM) for 24 h. The morphological changes of nuclei were examined using Hoechst 33342 staining. Scale bars: 100 µm. The blowup images from the red boxes show nuclear condensation and structural changes after exposure to anlotinib. (B and D) Effect of anlotinib on mitotic spindle formation in thyroid cancer cells. Cells were treated with or without 10 µM anlotinib for 24 h and subjected to immunofluorescence staining for TUBULIN. The right plots show the percentage of cells with abnormal spindle in the metaphase cells treated with 0, 1, 5 and 10 µM anlotinib. Data are shown as mean ± s.d. (*n* ≥ 3).

To address how anlotinib arrests cells at the G2/M phase, we checked the morphology of nuclei in BCPAP and 8505C cells with or without anlotinib treatment for 24 h. A significant fraction of nuclei in anlotinib-treated cells become condensed and fragmented, while the control cells without anlotinib treatment exhibit normal intact nuclei (Fig. 3A and C). Further examination of the spindle microtubule organization in mitotic cells revealed that anlotinib causes abnormal spindle organization. Rather than di-polar spindles, tri-polar spindles were observed in anlotinib-treated mitotic cells. The percentage of mitotic cells with abnormal spindles increases in an anlotinib dose-dependent manner (Fig. 3B and D). These data suggested that deregulated spindle formation by anlotinib treatment might account for the G2/M phase arrest of thyroid cancer cells.

### Anlotinib induces apoptosis in thyroid cancer cells partly through activating the TP53 pathway

**Figure 4:**
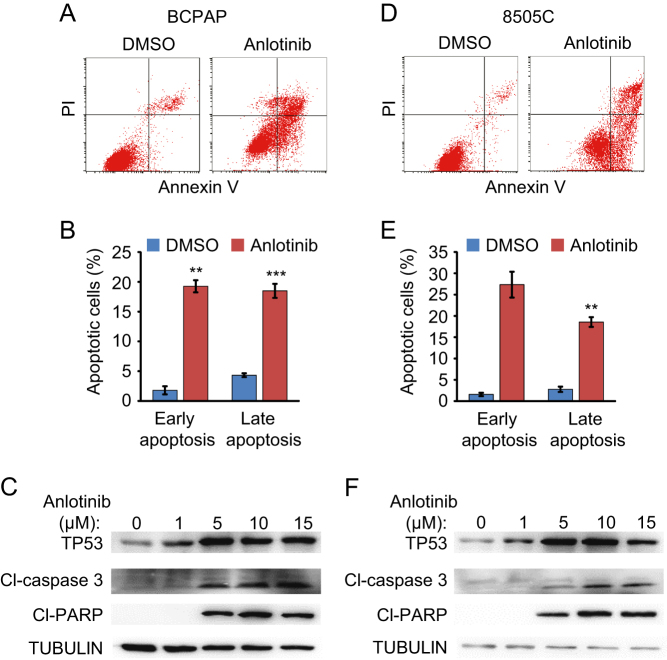
Anlotinib induces apoptosis in thyroid cancer cells. (A and D) BCPAP (A) and 8505C (D) cells were treated with DMSO and 10 µM anlotinib for 36 h. Cells were stained with Annexin V-FITC/PI and analyzed by flow cytometry. The low right (LR) quadrant of the histograms indicates the early apoptotic cells, and the upper right quadrant indicates the late apoptotic cells. (B and E) Quantification results of experiments described in A and D. Data are shown as mean ± s.d. (*n* ≥ 3). (C and F) BCPAP (C) and 8505C (F) cells were treated with anlotinib (0, 1, 5, 10 and 15 µM) for 36 h. Cell lysates were prepared and subjected to Western blot to detect the expression of TP53, cleaved caspase 3 (CL-caspase 3) and cleaved PARP (CL-PARP).

We have observed anlotinib-induced thyroid cancer cell apoptosis in cell cycle analysis (Fig. 2). To further confirm the apoptosis induced by anlotinib, BCPAP and 8505C cells, with or without anlotinib treatment for 36 h, were stained with Annexin V-FITC/PI and analyzed by flow cytometry. The results demonstrated that anlotinib treatment induces apoptosis in both BCPAP and 8050C cells. The fractions of early and late apoptotic anlotinib-treated BCPAP cells are 19.3 and 19.2%, respectively. And 27.3 and 18.6% of anlotinib-treated 8050C cells are in the early and late apoptosis, respectively (Fig. 4A, B, D and E). These results further validated that anlotinib stimulates the apoptosis of thyroid cancer cells.

In order to investigate the mechanism how anlotinib induces apoptosis in thyroid cancer cells, the expression levels of apoptosis-related proteins, including TP53, cleaved caspase 3 and cleaved PARP, were examined by Western blot. The results revealed that anlotinib upregulates the expression of TP53, as well as apoptotic markers cleaved caspase 3 and cleaved PARP, in a dose-dependent manner (Fig. 4C and F). Therefore, anlotinib might induce the apoptosis of thyroid cancer cells through activating the TP53 pathway.

**Figure 5:**
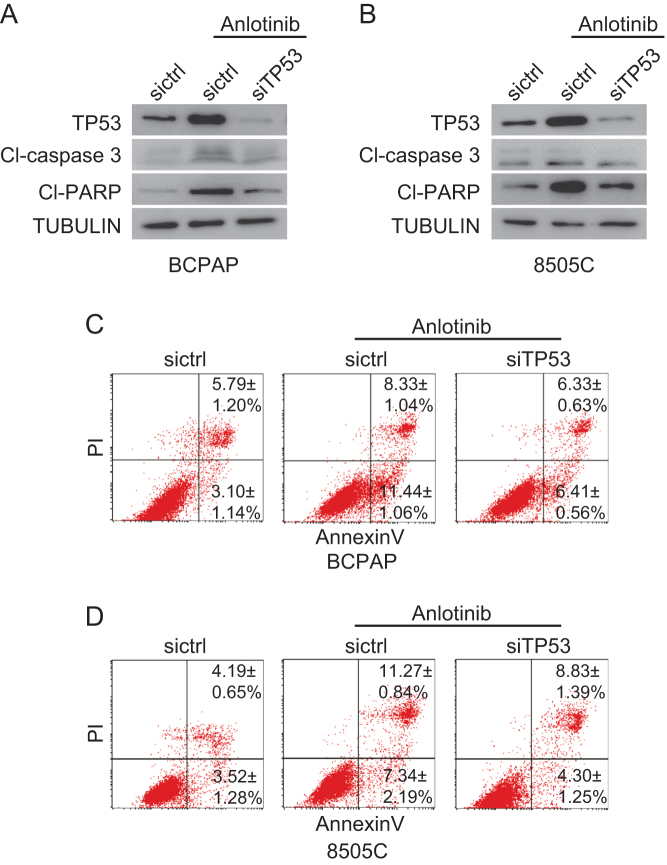
Anlotinib induces apoptosis partly through activating the TP53 pathway. (A and B) siRNA target TP53 and control RNA were transfected into thyroid cancer cells. Twenty-four hours after transfection, the cells were treated with or without 10 µM anlotinib for 24 h. Cell lysates were prepared and subjected to Western blot. (C and D) Thyroid cancer cells described in (A and B) were stained with Annexin V-FITC/PI and analyzed with flow cytometry. Data are shown as mean ± s.d. (*n* ≥ 3).

To confirm the role of TP53 in the apoptosis induced by anlotinib, TP53 was knocked down by siRNA in thyroid cancer cells. After transfection with the control RNA and siRNA, the cells were treated with 10 µM anlotinib for 24 h. Compared with the control cells transfected with siRNA, the levels of apoptotic markers cleaved caspase 3 and cleaved PARP in TP53-knockdown thyroid cancer cells are reduced (Fig. 5A and B). Consistently, few TP53-knockdown thyroid cancer cells undergo apoptosis upon anlotinib treatment (Fig. 5C and D). These data suggested that the TP53 pathway at least partly, if not entirely, mediates the apoptosis inducing effect of anlotinib.

### Anlotinib inhibits the migration of thyroid cancer cells

**Figure 6:**
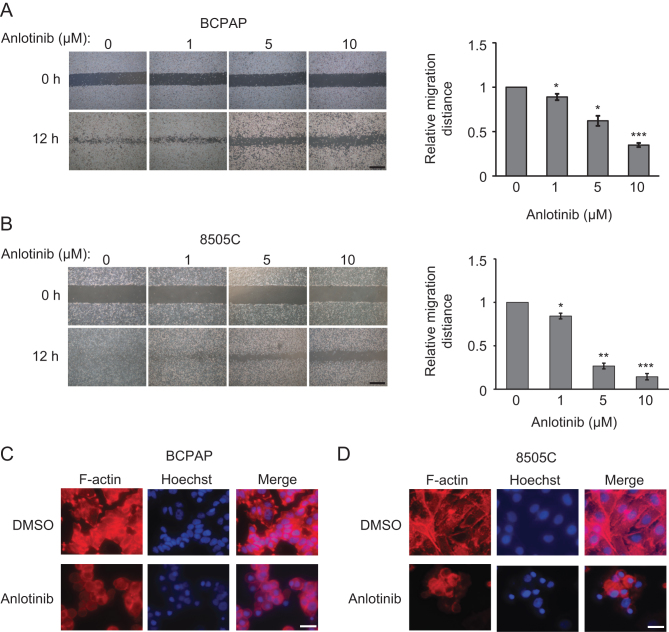
The effects of anlotinib on the migration of thyroid cancer cells. (A and B) Representative images of wound-healing assays using BCPAP (A) and 8505C (B) cells in the presence of 0, 1, 5 and 10 µM anlotinib. Scale bars: 25 µm. The plots on the right show the quantification of wound healing assays. Data are shown as mean ± s.d. (*n* ≥ 3). (C and D) The expression and cellular distribution of F-actin in BCPAP (C) and 8505C cells (D). Cells were treated with DMSO or 10 µM anlotinib for 24 h, and then stained with rhodamine-phalloidin (red) for F-actin. Hoechst 33342 was applied to counter-stain the nucleus (blue). Scale bars: 100 µm.

To investigate whether anlotinib impairs the migration of thyroid cancer cells, we performed wound-healing assay. We found that anlotinib dose dependently decreased the migration ability of BCPAP and 8505C cells. The wound closure rates of anlotinib-treated cells were lower than those of non-treated cells (Fig. 6A and B).

Since cell migration depends on actin filament (F-actin) remodeling, we tested the hypothesis that anlotinib inhibits thyroid cancer cell migration by interfering F-actin organization. F-actin was stained with rhodamine-phalloidin in both BCPAP and 8505C cells with or without anlotinib treatment. It is notable that anlotinib treatment alters the organization of F-actin in BCPAP and 8505C cells. Control untreated cells are elongated, with one or two predominant lamellipodia per cell. In anlotinib-treated cells, less F-actin is formed, and the lamellipodia nearly disappeared (Fig. 6C and D). These data suggested that anlotinib might interfere F-actin formation to suppress cell migration.

### Anlotinib suppresses thyroid tumor growth *in vivo*

**Figure 7:**
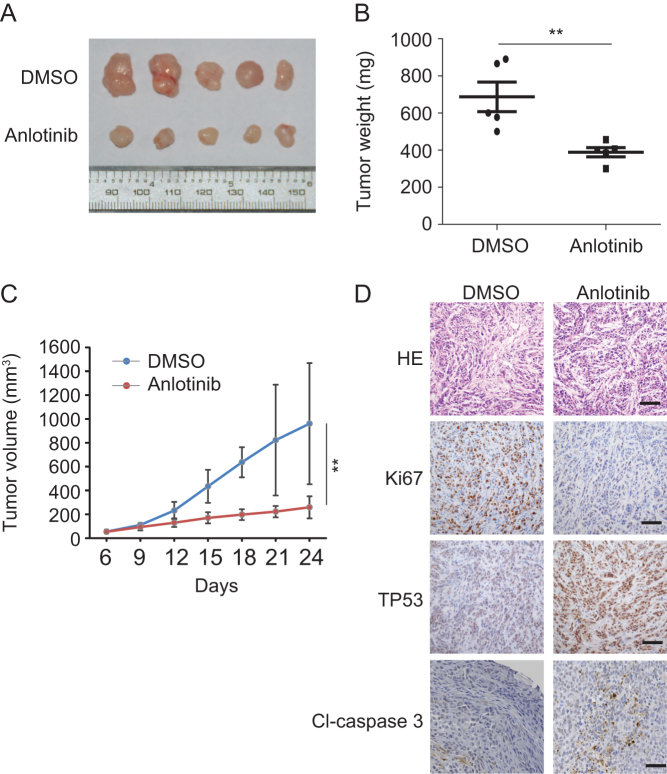
Anlotinib suppresses tumor growth *in vivo*. (A) Images of dissected tumors from nude mice injected with K1 cells of DMSO treated group (*n* = 5) and anlotinib treated group (*n* = 5). (B) Tumor weights of control and anlotinib-treated groups. (C) Tumor volumes with or without anlotinib treatment. (D) HE staining, Ki67, TP53 and CL-caspase 3 IHC staining of the tumors with or without anlotinib treatment. Scale bars: 100 µm.

Next, we used a K1 xenograft tumor model to determine whether anlotinib inhibits thyroid cancer growth *in vivo*. The anlotinib-treated group started to exhibit reduced tumor growth on day 9 and showed significantly decreased tumor weight and tumor volume at the end of the experiment (Fig. 7A, B and C). Moreover, anlotinib had little effect on bodyweight in mice during the course of the experiment (Supplementary Fig. 3). Compared with control group, anlotinib-treated group had fewer Ki67-positive cells, meaning fewer proliferating cells (Fig. 7D). Consistent with cell-based assay (Figs 4 and 5), anlotinib treatment also elevates the expression level of TP53 and cleaved caspase 3 in thyroid cancer xenografts (Fig. 7D), implying that apoptosis might be induced in these anlotinib-treated thyroid cancer xenografts. These data demonstrated that anlotinib inhibits thyroid tumor growth *in vivo*.

## Discussion

In most well-differentiated thyroid cancer cases, patients have excellent prognosis, whereas a minority of well-differentiated thyroid cancer patients die due to locoregional recurrence and distant metastasis (Zhang* et al.* 2016). However, ATC is extremely aggressive, and the median survival of ATC patients is only 3–5 months after diagnosis (Machens* et al.* 2005). The development of novel effective therapeutic agents for thyroid cancer, particularly for ATC, remains an urgent medical requirement. In this study, we investigated the potential therapeutic effect of a novel multi-kinase inhibitor anlotinib on thyroid cancers. Our data suggested that anlotinib has antitumor effect for both PTC and ATC cells. The IC_50_ values of anlotinib for the six PTC or ATC cell lines used in the present study range from 3.02 µM to 5.42 µM. Moreover, daily intraperitoneal treatment of 3 mg/kg anlotinib significantly suppressed tumor growth in the thyroid cancer xenograft model. These data provide the foundation of dosage for future clinical trials.

Several mechanisms may contribute to the antitumor effect of anlotinib. First, anlotinib treatment compromises the proper organization of mitotic spindle, leading to G2/M arrest and a slower proliferation rate. Second, anlotinib elevates the expression of TP53, thus inducing cell apoptosis. Third, anlotinib prevents cell migration, likely through interfering F-actin formation.

Interestingly, both BCPAP and 8505C cells harbor TP53 mutations, D259Y and R248G, respectively (Saiselet *et al.* 2012). Nevertheless, TP53 still mediates the proapoptotic effect of anlotinib in these two cell lines. There are at least two possible reasons for this phenomenon. D259Y and R248G mutations affect the DNA-binding activity of TP53, thus resulting in loss of function of TP53. Nevertheless, these mutations are different from null TP53 mutation. The DNA-binding ability of D259Y and R248G TP53 mutants can be reactivated by a compound, named TP53 reactivation and induction of massive apoptosis (PRIMA-1) (Rehman *et al.* 2005, Messina *et al.* 2012). Thus, anlotinib might be able to reactivate D259Y and R248G TP53 mutants. Alternatively, the transcription-independent function of TP53 might account for its proapoptotic effect in D259Y and R248G TP53-mutant cells. Overexpression of a mutant TP53, lacking most of the DNA-binding domain and completely deficient in transactivation function, could trigger apoptosis (Haupt *et al.* 1995, Green & Kroemer 2009). Similarly, activation of TP53 was found to trigger apoptosis even in the absence of a nucleus (Chipuk *et al.* 2003). Thus, D259Y and R248G TP53 mutants are able to induce apoptosis through its transcription-independent function.

However, the direct target(s) of anlotinib mediating its therapeutic effect on thyroid cancers remain elusive. Despite its important role in the pathogenesis of thyroid cancer (Murugan* et al.* 2009), the BRAF/MEK/ERK signaling pathway is unlikely the key downstream target of anlotinib. First, anlotinib suppresses the BRAF/MEK/ERK pathway only in BCPAP cells, but not in 8050C cells, while anlotinib effectively inhibits the proliferation of both cell lines. Second, inactivation of the BRAF/MEK/ERK pathway by a MEK inhibitor PD0325901 slows down the proliferation of BCPAP slightly, but not 8050C cells (Supplementary Fig. 2). Future efforts are required to identify the direct target(s) of anlotinib responsible for its anti-proliferation, pro-apoptosis and anti-migration effects. With this knowledge, drug with better specificity and less side-effect may be developed.

In summary, our data demonstrated that anlotinib exerts antitumor effects on thyroid cancer cells through inhibition of cell growth, induction of apoptosis and suppression of cell migration. Importantly, anlotinib is effective against various thyroid carcinoma cell lines, including both PTC and ATC. Importantly, anlotinib also suppresses thyroid tumor growth *in vivo*. Thus, anlotinib could be developed as a novel effective antitumor agent for thyroid cancer treatment, especially for poorly differentiated papillary thyroid cancers and ATC, for which no effective therapeutic treatment is available.

## Supplementary Material

Supporting Figure 1

Supporting Figure 2

Supporting Figure 3

## Declaration of interest

The authors declare that there is no conflict of interest that could be perceived as prejudicing the impartiality of the research reported.

## Funding

This work did not receive any specific grant from any funding agency in the public, commercial, or not-for-profit sector.

## Author contribution statement

X R, X S, Q D, Y Y, X H, X W and X S performed the experiments, M G and L C conceived the experiments and wrote the manuscript.
