# Supplementary figures and images for: Antitumor effects of anlotinib in thyroid cancer
Source: Endocr Relat Cancer. 2018 Aug 21;26(1):153–64. doi: 10.1530/ERC-17-0558 (PMC6215907; doi:10.1530/ERC-17-0558)

A

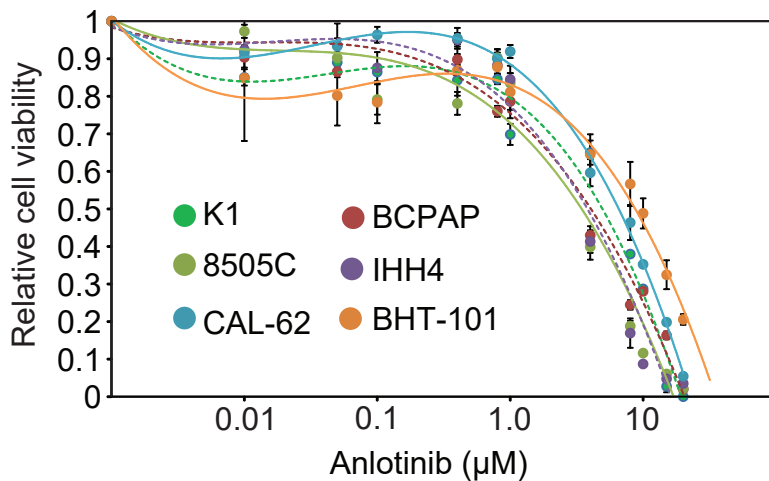

B

| Cell line | Type | IC50 (μM) |
|-----------|------|-----------|
| BCPAP     | PTC  | 3.02      |
| K1        | PTC  | 3.97      |
| IHH4      | PTC  | 3.06      |
| 8505C     | ATC  | 3.18      |
| CAL-62    | ATC  | 4.64      |
| BHT-101   | ATC  | 5.42      |

Supplement: Supporting Figure 1 [file erc-26-153-s001.pdf]

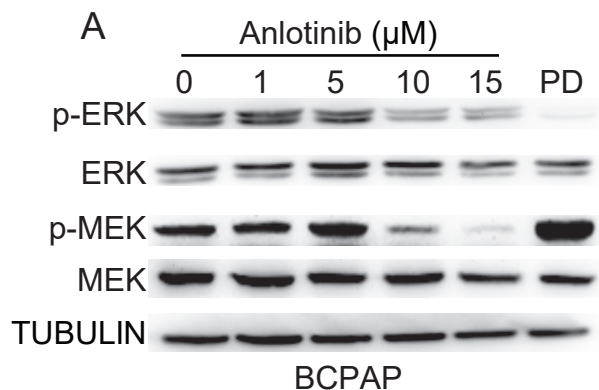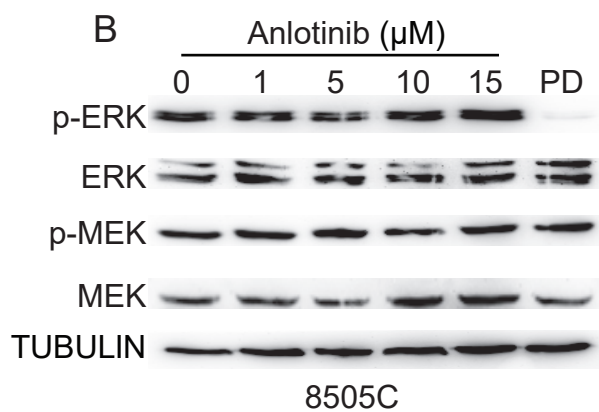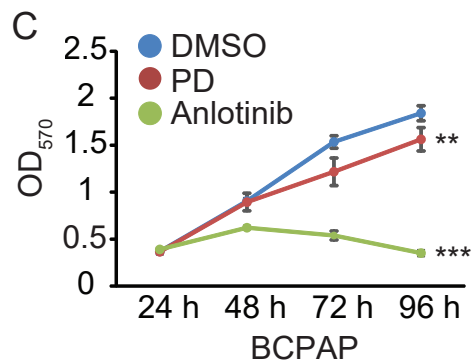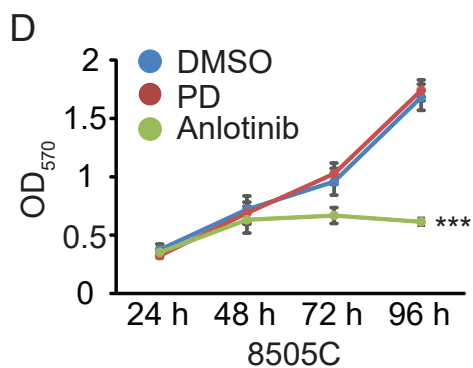

Supplement: Supporting Figure 2 [file erc-26-153-s002.pdf]

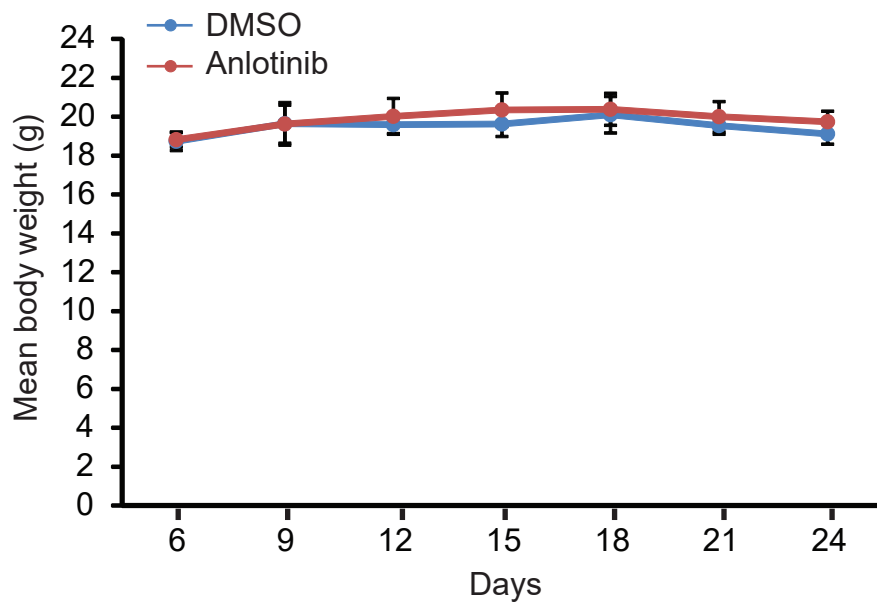

Supplement: Supporting Figure 3 [file erc-26-153-s003.pdf]
